# Do we need CBCTs for sufficient diagnostics?-dentist-related factors

**DOI:** 10.1186/s40729-018-0147-1

**Published:** 2018-11-16

**Authors:** Josipa Radic, Raphael Patcas, Bernd Stadlinger, Daniel Wiedemeier, Martin Rücker, Barbara Giacomelli-Hiestand

**Affiliations:** 1 Clinic of Cranio-Maxillofacial and Oral Surgery, Centre of Dental Medicine, University of Zurich, Plattenstrasse 11, 8032 Zurich, Switzerland; 2 Clinic for Orthodontics and Paediatric Dentistry, Centre of Dental Medicine, University of Zurich, Plattenstrasse 11, 8032 Zurich, Switzerland; 3 Statistical Services, Centre of Dental Medicine, University of Zurich, Plattenstrasse 11, 8032 Zurich, Switzerland

**Keywords:** Anatomy, CBCT, OPT, Oral surgery, Orthodontics

## Abstract

**Background:**

The aim of this study was to assess the diagnostic accuracy of various dentoalveolar pathologies based on panoramic radiography (OPG), cone beam computed tomography (CBCT) and printed 3D models in consecutive order; and to evaluate the impact of specialisation of residents in oral surgery (OS) versus residents in orthodontics (ORTH).

**Methods:**

Fourteen residents were recruited to evaluate nine selected cases with different dentoalveolar pathologies. The residents were given for each case an OPG, a CBCT and a printed 3D model. For each case and imaging modality, the residents were asked several questions relating to (i) diagnosis, and (ii) the request for consecutive imaging in order to enable treatment. Further, aspects like impact of specialisation (OS versus ORTH), gender and years of experience were analysed.

**Results:**

In this study, diagnostic accuracy (i) improved for OS from OPG to CBCT (OPG 66.3%, CBCT 83.4%) and likewise for ORTH (OPG 63.7%, CBCT 78.0%). 3D models generally did not seem more useful than CBCTs. For treatment planning (ii), residents in orthodontics considered OPGs significantly more often as sufficient compared to residents in oral surgery (OR 6.3, *p* < 0.001). Further, the odds to request a CBCT after OPG for treatment planning is influenced by dentist-related factors: female dentists (OR 3.8) or residents with limited professional experience as dentists (OR 3.0) asked more frequently for a CBCT.

**Conclusions:**

Overall diagnostic accuracy is decent with OPG and can be improved with CBCT. Specialisation seems to have a moderate impact on diagnostic accuracy, but influences whether a CBCT was requested for treatment planning. Based on these findings, future studies shall analyse the diagnostic accuracy of specific pathologies in higher number in order to substantiate the present findings with regard to specific pathologies.

## Background

Along with the clinical examination, radiological imaging is essential for a complete diagnosis in dental medicine [1, 2]. Orthopantomography (OPG), a two-dimensional panoramic radiograph, is widely used across all dental disciplines including oral surgery and orthodontics [3–5] to address basic diagnostic queries. An OPG contains an abundance of information on the teeth, mandible, maxilla, including the sinuses and the nasal cavity, and the temporomandibular joints. At the same time, OPG images suffer from important limitations [4, 6], such as being limited to two dimensions, distortion and blurring. In some cases, the broad coverage of the OPG will therefore still be insufficient to obtain an accurate diagnosis or enable the dentist to perform a treatment plan. According to most prevailing guidelines [7–9], three-dimensional imaging is recommended for patients who will benefit from cone beam computed tomography (CBCT) because the diagnosis would otherwise remain uncertain or the treatment plan unclear. CBCT scans of a region of interest have particularly been advocated by these guidelines for the assessment of bony structures and teeth, trauma, various pathologies or the assessment of topographical anatomy prior to surgical or orthodontic procedures.

While the guidelines are hardly disputed, it must be noted that diagnostic accuracy and the effect on patient management (such as setting a treatment plan) are two different facets of imaging efficacy and have historically always been viewed in a hierarchical order [10, 11]. Moreover, an intrinsic difference between diagnostic accuracy and patient management must be respected. While diagnosis of a pathology should clearly remain unaffected by the dental specialisations, the treatment plan may ultimately differ between disciplines. The evaluation of a CBCT scan should therefore preferably include the impact on diagnostic accuracy as well as treatment approach.

With the introduction of commercial 3D-printing in dentistry, another diagnostic tool became available. The printing of CBCT DICOM-based surface reconstructed files with a semi-opaque material enables to obtain a “see-through” physical model of the scan. Yet, the diagnostic value of such printed models has to our knowledge not been researched.

Finally, the request for a CBCT should always be guided by the pursuit of improved diagnostic accuracy and the prospect of an enhanced treatment plan. Preferably, the indications for a CBCT should be based entirely on case-related factors. Yet, dentist-related factors might influence the request for a CBCT as well.

The aim of this study was therefore (i) to assess whether pathologies are accurately diagnosed in three different imaging modalities (OPG, CBCT, 3D model) of the same case, and (ii) whether the case is classified as treatable on the basis of the present imaging modality. Further, aspects like the impact of specialisation (oral surgery versus orthodontics), gender and years as a dentist were analysed.

## Methods

Fourteen residents were recruited for this survey [7 residents in oral surgery (OS) and 7 residents in orthodontics (ORTH), respectively; *m* = 6, *f* = 8]. Their characteristics are listed in Table 1. Every resident assessed individually nine separate patient cases, each containing a distinct dentoalveolar pathology, as defined in the study planning process (Table 2).

**Table 1:** Characteristics of residents in oral surgery and orthodontics

Resident	Age (years)	Sex	Specialisation	Experience as a dentist (years)
1	31	m	OS	3 to 4
2	29	m	ORTH	3 to 4
3	34	m	ORTH	5 to 9
4	30	m	ORTH	5 to 9
5	29	f	OS	3 to 4
6	30	f	ORTH	3 to 4
7	34	m	ORTH	5 to 9
8	27	f	OS	1 to 2
9	28	f	ORTH	3 to 4
10	28	f	ORTH	1 to 2
11	29	f	OS	3 to 4
12	29	f	OS	1 to 2
13	28	f	OS	1 to 2
14	31	m	OS	3 to 4

*m*, male; *f*, female; *OS*, oral surgery; *ORTH*, orthodontics

**Table 2:** Description of the cases assessed

Case	Age (years)	Sex	Pathology	Time between OPG and CBCT
1	28	m	Tooth resorption	0 Mt
2	11	f	Retained tooth	3 Mt
3	14	m	Retained tooth	5 Mt
4	13	m	Mesiodens	1 Mt
5	18	f	Retained tooth	1 Mt
6	12	m	Tooth resorption	2 Mt
7	13	f	Retained tooth	1 Mt
8	17	m	Odontoma	0 Mt
9	13	f	Retained tooth	0 Mt

*m*, male; *f*, female; *Mt*, months

For each patient case, an OPG, a CBCT and a printed 3D model were shown in sequential order to each resident. Each resident assessed each patient case and each type of imaging modality (OPG, CBCT, 3D model) on the basis of a questionnaire customised for this study. The questionnaire comprised diagnostic items and questions relating to diagnosis and patient management (treatability). The correct diagnosis of each case was determined by two independent and experienced senior consultants prior to the residents’ assessment.

Medical records of the Clinic of Cranio-Maxillofacial and Oral Surgery and the Clinic of Orthodontics and Paediatric Dentistry of the University of Zurich, Switzerland were searched for patient cases with the following inclusion criteria:

- Treatment indication due to supernumerary, displaced or retained teeth, bone or teeth resorptions, or odontomas
- Availability of an OPG and a CBCT (Accuitomo 170)
- Patient age between 6 and 30 years
- Time lapse between OPG and CBCT no more than 6 months, no dental treatment performed between the imaging
- Informed consent for further use of patient data for clinical research given by the patient and wherever necessary by the parents/legal guardians

Every resident assessed the cases separately in the presence of the same supervisor, in order to ascertain identical settings and equal instructions to the viewer software (Morita I-Dixel). Uncertainties were clarified prior to completing the questionnaires. Patient cases were anonymised and presented to every resident in the same order.

The residents had to answer the questions under the following standardised conditions:

- Time available: 1 h 15 min
- Each resident assessed three images per patient in the following sequence: OPG CBCT, 3D model. For each image modality, a questionnaire had to be filled out
- Each resident was shown the region of interest to which the questions related to
- Allowed setup change of OPG: zoom
- Allowed setup change of CBCT: brightness, contrast, zoom, scroll in all three levels (coronal, axial and sagittal
- 3D model: no restrictions

The OPGs of this study were taken either in-house (CRANEX D, Kw73, 10 mA) or extramural. All CBCTs were taken at the Centre of Dental Medicine of the University of Zurich (CBCT: 3D-Accuitomo 170). In order to produce printed 3D models, DICOM data of the region of interest were cropped and STL-files produced using dedicated software (Slicer 4.5.0.) The STL file was printed with a 3D printer (Objet Eden 260 V) in a resolution of 600 dpi with a horizontal layering of 16 μm, using a semi-translucent material (synthetic material, Med610 Stratasys).

For each case and image, the following nine categorical items had to be answered (yes; no; available information not sufficient):

1. Is there a direct contact between teeth/tooth structures and nerve structures?
2. Is the tooth displaced?
3. Is there more than one root?
4. Is there a direct non-physiological contact between the tooth/tooth structure and the adjacent teeth?
5. Is there a pericoronal cyst formation, respectively a cystic formation originating from the tooth/tooth structure?
6. Has more than a third of the root maturation been completed?
7. Is a resorption in the bone or tooth structure visible?
8. Is an ankylosis visible?
9. Is the tooth/tooth structure worth being preserved?

Following questions relating to the treatability were asked after the OPG evaluation:

10. Is this case treatable with this amount of information?
11. Would you request further imaging to improve your diagnostic accuracy?

Even if in the resident’s opinion the OPG provided sufficient information to answer all questions, the resident was nevertheless requested to assess the CBCT. The same applied to the 3D model. After every imaging modality assessment, the questionnaires were collected to avoid retrospect changes.

### Statistical analysis

Statistical analysis and plots were performed using the statistical software R [12]. To evaluate the differences in the proportions of correct diagnostic answers between OS and ORTH and between different imaging modalities, Fisher’s exact tests were used and odds ratios (OR) including confidence intervals (CI) were computed for every question separately. Likewise, Fisher’s exact tests were applied to estimate whether there was a difference between the answers given by OS and ORTH on the question if the image provided sufficient information to treat the case (treatability).The same test procedure was also used to investigate if a CBCT after OPG was requested with regard to treatment planning and if specialisation, gender and years of experience as a dentist (dichotomized in 0–4 years of experience versus more) were associated with it. Statistical significance was set to α = 0.05 for all analysis.

## Results

### Diagnostic accuracy (i)

**Fig. 1:**
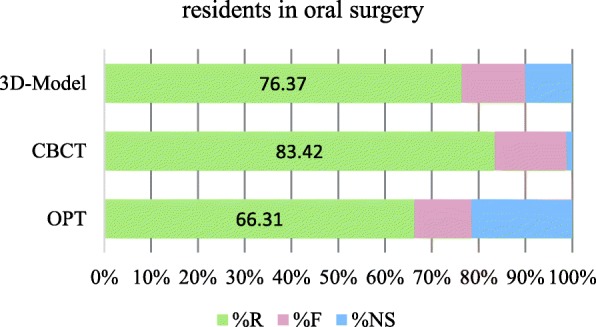
Accuracy of diagnostic answers from residents in oral surgery (R right, F false, NS not sufficient)

**Fig. 2:**
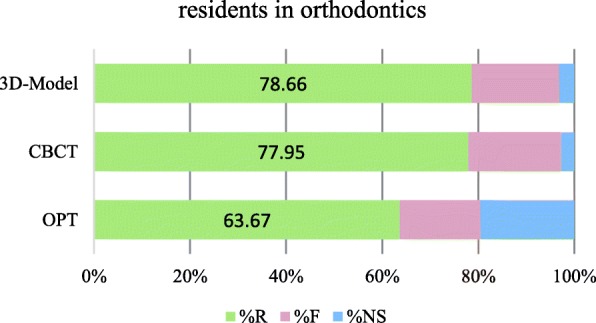
Accuracy of diagnostic answers given by residents in orthodontics (R right, F false, NS not sufficient)

Overall, the majority of the questions were answered correctly, independently to the imaging modality. The percentages of correct answers given by OS were 66.3% for OPG, 83.4% for CBCT and 76.4% for 3D model; and differed slightly to those given by ORTH with 63.7% for OPG, 78.0% for CBCT and 78.7% for 3D model (Figs. 1 and 2). Both OS and ORTH alike answered to around 20% of the questions that the OPG provided insufficient data in order to answer the question.

Assessing a CBCT increased the percentage of correct answers after OPG assessment, both for OS and ORTH. When given a printed 3D model after CBCT, an additional increase in correct answers could be observed for ORTH, but not for OS (Fig. 2 versus Fig. 1).

Evaluating the different questions independently, only three questions were answered significantly different between OS and ORTH (Table 3). The evaluation of a contact between tooth/tooth structure and nerve, the appraisal of root maturation and the diagnosis of a resorption reached in the case of this study higher percentage for the OS group, with odds ratios varying between 1.7 and 3.8. Otherwise, no apparent differences could be detected in the correctness of the answers.

**Table 3:** Accuracy of the diagnostic answers given, according to specialisation: residents in oral surgery versus residents in orthodontics

Question pertaining to	OS (%)	ORTH (%)	*p* value	Odds ratio (95% CI)
Contact to nerve	96	87	0.002*	3.8 (1.4–12.0)
Displacement of tooth	92	87	0.089	1.9 (0.9–4.1)
Number of roots	95	95	1.000	–
Contact to adjacent teeth	74	71	0.626	1.1 (0.7–1.9)
Pericoronar cyst	77	72	0.323	1.3 (0.8–2.2)
Maturation of root	89	80	0.029*	2.0 (1.1–3.7)
Resorption (bone or tooth)	76	65	0.040*	1.7 (1.0–2.8)
Ankylosis	83	89	0.176	0.6 (0.3–1.3)
Preservation	79	80	0.793	0.9 (0.5–1.6)

Percentage of correct answers given by residents in oral surgery (OS) and residents in orthodontics (ORTH). To assess the differences between the specialisations, *p* value of Fisher’s exact test is given together with odds ratio (OR), including 95% confidence intervals (CI). OR refers to OS with ORTH as reference. *p < 0.05

When comparing the diagnostic accuracy based on OPG and CBCT (following OPG), no differences could be observed for any of the nine questions (Table 4). The same was true when assessing impact of age, gender and years of experience as a dentist on the accuracy of the answers.

**Table 4:** Accuracy of the diagnostic answers given, according to imaging modality: OPG versus CBCT

Question pertaining to	OPG (%)	CBCT (%)	*p* value	Odds ratio (95% CI)
Contact to nerve	91	93	0.616	0.8 (0.3–2.4)
Displacement of tooth	88	90	0.543	0.7 (0.3–0.5)
Number of roots	95	94	0.775	1.2 (0.3–5.1)
Contact to adjacent teeth	73	70	0.643	1.2 (0.6–2.3)
Pericoronar cyst	73	75	0.764	0.9 (0.5–1.7)
Maturation of root	84	83	1.000	–
Resorption (bone or tooth) toothtooth)	65	72	0.305	0.7 (0.4–1.4)
Ankylosis	86	85	1.000	–
Preservation	83	78	0.510	1.3 (0.6–2.7)

Percentage of correct answers given based on OPG and CBCT (after OPG). To assess the differences between the imaging modality, *p* value of Fisher’s exact test is given together with odds ratio (OR), including 95% confidence intervals (CI). OR refers to CBCT with OPG as reference

### Treatability (ii)

**Fig. 3:**
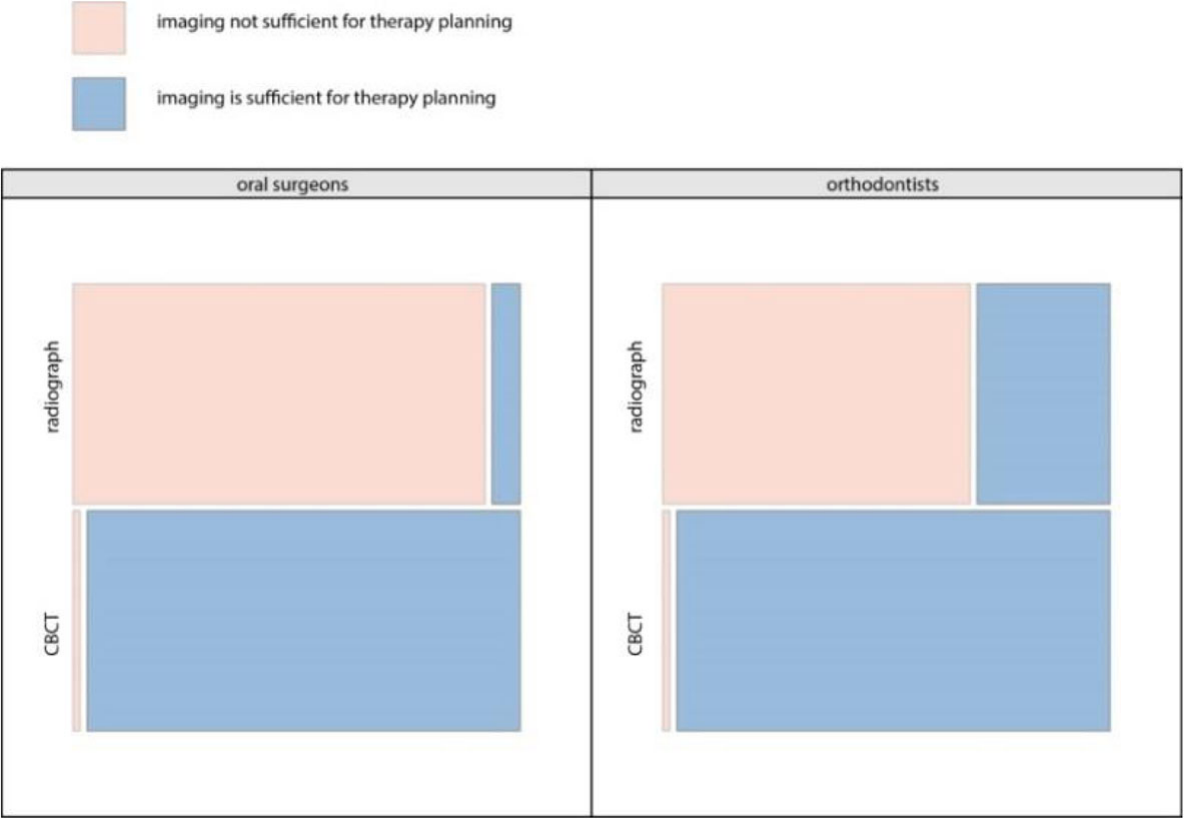
Treatability refers to OPG/CBCT and to residents in oral surgery and orthodontics

At the end of every OPG evaluation, residents were asked whether further imaging was deemed necessary in order to improve diagnostic accuracy. In 81.6% of the cases, further imaging was requested after the OPG. When asked whether the image assessed provided sufficient information to enable a treatment, the ORTH considered the information content of OPGs significantly more often as sufficient compared to the OS group (*p* < 0.05) (Fig. 3). This decision was highly influenced by the residents’ background. The odds to request a CBCT were far greater if the OPG assessment was done by a OS (OR 6.3), by a female dentist (OR 3.8) or dentists with only 0 to 4 year of experience (OR 3.0) (Table 5).

**Table 5:** Request of CBCT after OPG: influence of residents’ characteristics

Variable	*p* value	Odds ratio (95% CI)
Specialisation: oral surgery vs orthodontics	< 0.001*	6.3 (1.9–27.2)
Gender: females vs males	0.005*	3.8 (1.4–12.2)
Years of professional experience: 0–4 years vs > 4 year	0.045*	3.0 (1.0–9.9)

*p* values of Fisher’s exact test is given together with odds ratio (OR), including 95% confidence intervals (CI). OR refers to the first listed outcome with the second listed outcome being used as reference. *p < 0.05

## Discussion

The aim of this study was twofold: (i) to analyse the diagnostic accuracy of pathologies in three different imaging modalities of the same case and (ii) to analyse the need for further imaging in order to enable treatment. Further, aspects like the impact of specialisation, gender and dental experience were analysed. In contrast to the plethora of scientific literature available dealing with CBCT image accuracy, not much research has been conducted on how CBCT data are being contextually handled. This present investigation was designed to increase our understanding in this specific area.

### Diagnostic accuracy (i)

The first objective was not only to analyse if diagnostic accuracy of residents is improved when assessing a CBCT (compared to an OPG), but to dissect the data and evaluate whether the diagnostic accuracy varies when assessing different pathologies, and whether professional background would account for the performance. Although a general increase of more correct answers could be observed for the assessment of CBCT compared to OPG (Figs. 1 and 2), the impact of CBCT was not present in every case when establishing the effect for each clinical question. Somewhat unexpectedly, comparable diagnostic accuracy could be attained already with an OPG for many diagnosed pathologies. Professional background had likewise a very small influence, as specialisation affected only the correctness of three answers moderately, and age, gender and years of experience as a dentist showed no effect at all. In short, only questions relating to resorption of dental or bony tissue, contact to nerve and maturation stage of the root reached significantly higher percentages for the residents in oral surgery group.

Comparable improvement in diagnostic accuracy was shown in previous studies [13] when comparing CBCT to OPG, and there is a broad consensus on the added value of CBCT imaging in diagnostics and treatment planning compared to a two-dimensional imaging [13–23]. Our results are in full agreement with these publications, yet highlight the fact that dental education may influence diagnostic accuracy, depending on the pathology to be assessed.

Moreover, another valuable and novel observation is the divergence seen in the importance of printed 3D models. For residents in oral surgery, printed 3D models caused more uncertainties and led to a decrease of diagnostic accuracy (if assessed in sequential order after OPG and CBCT). In contrast, residents in orthodontics seemed to benefit of an additional assessment of printed 3D models, which resulted in an improvement of their diagnostic ability. This increase in diagnostic accuracy might be partially explained with the larger experience residents in orthodontics share with model assessment.

### Treatability (ii)

All residents were asked whether the OPG contained sufficient information to enable a treatment. Interestingly, most residents in orthodontics and nearly all residents in oral surgery stated that the OPG did not provide sufficient information for a treatment plan (Fig. 3), even though the majority of the diagnoses were done correctly. Apparently, this dissonance indicates that diagnostic content cannot be equated with the required information needed for treatability. An accurate diagnosis is an essential part of treatment planning, but clinicians obviously look out for more than just a diagnosis when they evaluate and interpret an OPG. The observation that the treatability of the cases is viewed differently after CBCT assessment is in full agreement to previous studies [17, 24, 25].

Perhaps rather surprisingly, residents in orthodontics were significantly more often satisfied with the information given by an OPG for their treatment planning than residents in oral surgery who stated that the case was not treatable with the information provided by an OPG. This trend is probably due to the different goals pursued by each specialisation. The questions that the residents in orthodontics would like to have answered by any imaging modality will probably differ from the queries that the residents in oral surgery aims to have solved. Even when the one and same case is assessed by a resident in oral surgery and orthodontics, every one of them will be focused to answer different questions relating to treatability. Hence, it may be that an OPG will contain enough information for the resident in orthodontist to pen his treatment plan, but may hold only insufficient data for queries related to a surgical approach.

In 81.6% of the cases, further imaging was requested after the OPG. Caution should be applied in the interpretation of this number, as the residents’ decision was theoretical and did not imply additional costs or radiation exposure. Nevertheless, it is striking that in the majority of the cases, further imaging was requested. One possible explanation might be the diagnostic difficulty of the chosen cases. This relevant finding indicates a high subjective demand for a CBCT and a lower objective necessity for further imaging after OPG, based on the present cases. However, this conclusion can only be drawn, after performing a CBCT. The onus rests on future scientific endeavours to find means to reduce this discrepancy.

The request for a CBCT was significantly influenced by the residents’ professional background (OS vs ORTH) and gender. Residents in oral surgery, female dentists and residents with limited professional experience as dentists indicated up to six times more often the need for further imaging. When analysing the assessment of impacted canines, Lai et al. observed similarly that oral surgeons requested more often CBCT imaging than orthodontists [19]. This might be explained by the fact that oral surgeons are interested in diagnostic information as well as in information for surgical planning. This takes positional relations with regard to surgical approaches into account, being facilitated by 3D imaging. As mentioned above, orthodontics and oral surgery differ in their judgement on treatability. This offers an obvious explanation for the difference regarding the request for further imaging. Residents in oral surgery, who stated more often that the cases were not treatable with the data obtained solely by OPG, were also the same group who requested significantly more CBCT, and vice versa. It is understandable that less experienced clinicians ask for more CBCT scans, but the interpretation why male dentist would request more CBCT scans is not trivial. Hodges et al. [17] demonstrated that clinicians who own CBCT devices requested more CBCT scans than other dentists. This and other reports suggest that dentist-related variables influence the request for CBCT scans at least as much as case-related factors.

Certain limitations affect the generalizability of this study’s results. First, only nine cases were assessed with a limited range of pathologies (five retained teeth (canines and molars), two tooth resorptions, one odontoma and one supernumerary tooth). Moreover, the assessment was performed by a small amount of residents of the local university. The fact that all residents shared a similar academic environment might be a source of inadvertent bias. Nevertheless, the odds of some of the portrayed observations are evident and far too important to be ignored on the pretext of the limitations.

## Conclusions

This study analysed (i) whether pathologies are accurately diagnosed in three different imaging modalities (OPG, CBCT, 3D model). Diagnostic accuracy was decent with OPG and was improved with CBCT. Next, the study assessed (ii) whether each case was classified as treatable on the basis of the present imaging modality. This result was influenced by the professional background, which influenced whether a CBCT was requested for treatment planning. Further, dentist-related factors like gender and the professional experience as a dentist also took an influence on the request for further imaging.

## Abbreviations

3D: Three-dimensional
CBCT: Cone beam computed tomography
DICOM: Digital Imaging and Communications in Medicine
OPG: Orthopantomography
OR: Odds ratio
ORTH: Resident in orthodontics
OS: Resident in oral surgery
